# 766 Battling Hyponatremia in Burn Patients

**DOI:** 10.1093/jbcr/irae036.308

**Published:** 2024-04-17

**Authors:** Michelle A Kaleita, Robert S Bush, Anthony Musalo, Meghan Bailey, Janet Popp, Ian R Driscoll, Andrea Munden

**Affiliations:** UF Health Shands, Gainesville, FL; UFHealth, Gainesville, FL; University of Florida College of Medicine, Gainesville, FL; Department of Surgery, University of Florida College of Medicine, Gainesville, FL; UF Health Shands, Gainesville, FL; UFHealth, Gainesville, FL; University of Florida College of Medicine, Gainesville, FL; Department of Surgery, University of Florida College of Medicine, Gainesville, FL; UF Health Shands, Gainesville, FL; UFHealth, Gainesville, FL; University of Florida College of Medicine, Gainesville, FL; Department of Surgery, University of Florida College of Medicine, Gainesville, FL; UF Health Shands, Gainesville, FL; UFHealth, Gainesville, FL; University of Florida College of Medicine, Gainesville, FL; Department of Surgery, University of Florida College of Medicine, Gainesville, FL; UF Health Shands, Gainesville, FL; UFHealth, Gainesville, FL; University of Florida College of Medicine, Gainesville, FL; Department of Surgery, University of Florida College of Medicine, Gainesville, FL; UF Health Shands, Gainesville, FL; UFHealth, Gainesville, FL; University of Florida College of Medicine, Gainesville, FL; Department of Surgery, University of Florida College of Medicine, Gainesville, FL; UF Health Shands, Gainesville, FL; UFHealth, Gainesville, FL; University of Florida College of Medicine, Gainesville, FL; Department of Surgery, University of Florida College of Medicine, Gainesville, FL

## Abstract

**Introduction:**

A trend of hyponatremia in burn patients prompted a data analysis of laboratory results which confirmed a need for improvement. A task force was formed to identify barriers. There was a knowledge deficit among staff related a free water restriction order. A literature search determined the recommended concentration of sodium per ounce to achieve eunatremia and suggested beverages. However, few palatable beverage choices were available. Popular electrolyte replacement beverages do not have sufficient sodium quantity per ounce so are considered free water. Pediatric electrolyte replacement beverages were not available to the adult population. A rapid rehydration beverage was discovered to have the appropriate ratio of sodium per ounce and came in various flavors.

Collaborated with Dietician, providers, and other departments experiencing similar issues. As a result, an order set was created listing appropriate sodium per ounce beverages and clarifying free water restriction guidelines. The rapid rehydration beverage was added to the Dietary floor stock. Staff were subsequently educated on the sodium per ounce requirement for a free water restriction, notably the difference between the popular electrolyte replacement beverage and the rapid rehydration beverage as well as the avoidance of water.

**Methods:**

• Pre-data analysis identified all sodium lab results from March 2022 - Feb 2023 which were under 135 mEq/L

• Fishbone diagram identified of contributing factors to hyponatremic

• Literature review of implementing free water restriction conducted

• Brainstormed ideas for alternative beverages

• Implemented interventions:

Educated staff on hyponatremia, free water restriction, and proper sodium/ounce

Eliminated water pitchers at the bedside

Created free water restriction door signs

Free water restriction order set created house-wide

• Post-data analysis of all sodium lab results from March 2023 - Aug 2023 which measured less than 135 mEq/L

**Results:**

Over a 12-month period, there were 5796 sodium results. Of those, 21.3% were below 135 mEq/L. After the interventions, there was a noticeable decrease in hyponatremia episodes. From March 2023 - Aug 2023, there were 3448 sodium results. Of those, 13.6% were below 135 mEq/L.

**Conclusions:**

The study shows that identifying issues contributing to hyponatremia and implementing proper measures can help decrease the episodes. Education, research, and finding a palatable rapid rehydration beverage can make a difference in sodium levels. Patients have been compliant with the new rapid rehydration beverage, and it makes it easier to keep patients willing to work with staff. Continuing research into this change could help stem new research in the future.

**Applicability of Research to Practice:**

Many things affect sodium and water metabolism and this has not been subjected yet to analysis, but will be in the future. Another consideration is to evaluate the rapid rehydration beverage as a resuscitation adjunct for smaller burns.

